# MRI Features in a Rat Model of H-ABC Tubulinopathy

**DOI:** 10.3389/fnins.2020.00555

**Published:** 2020-06-03

**Authors:** Angeles Garduno-Robles, Milvia Alata, Valeria Piazza, Carmen Cortes, Jose R. Eguibar, Sergio Pantano, Victor H. Hernandez

**Affiliations:** ^1^Departament of Chemical, Electronic and Biomedical Engineering, DCI, University of Guanajuato, Guanajuato, Mexico; ^2^Center of Research in Optics, Leon, Mexico; ^3^Institute of Physiology, Benemerita Universidad Autonoma de Puebla, Puebla, Mexico; ^4^Research Office of the Vice-rectory of Research and Postgraduate Studies, Benemerita Universidad Autonoma de Puebla, Puebla, Mexico; ^5^Group of Biomolecular Simulations, Institut Pasteur de Montevideo, Montevideo, Uruguay

**Keywords:** hypo/demyelination, tubulinopathies, H-ABC, *taiep*, MRI, optical microscopy, leukodystrophy

## Abstract

Tubulinopathies are a group of recently described diseases characterized by mutations in the tubulin genes. Mutations in *TUBB4A* produce diseases such as dystonia type 4 (DYT4) and hypomyelination with atrophy of the basal ganglia and cerebellum (H-ABC), which are clinically diagnosed by magnetic resonance imaging (MRI). We propose the *taiep* rat as the first animal model for tubulinopathies. The spontaneous mutant suffers from a syndrome related to a central leukodystrophy and characterized by tremor, ataxia, immobility, epilepsy, and paralysis. The pathological signs presented by these rats and the morphological changes we found by our longitudinal MRI study are similar to those of patients with mutations in *TUBB4A*. The diffuse atrophy we found in brain, cerebellum and spinal cord is related to the changes detectable in many human tubulinopathies and in particular in H-ABC patients, where myelin degeneration at the level of putamen and cerebellum is a clinical trademark of the disease. We performed *Tubb4a* exon analysis to corroborate the genetic defect and formulated hypotheses about the effect of amino acid 302 change on protein physiology. Optical microscopy of *taiep* rat cerebella and spinal cord confirmed the optical density loss in white matter associated with myelin loss, despite the persistence of neural fibers.

## Introduction

Tubulin is present in all nucleated cells, and at least three prokaryotic homologs are also known (Kim, 2019); in humans, the tubulin superfamily includes more than 20 genes (HUGO, 2020). The protein has a very conserved sequence and, when dimers of tubulin assemble into microtubules, they endow cells with a scaffolding for their shape and intracellular movements. In most eukaryotic organisms, different cell types and tissues express one or more tubulin isoforms or isotypes (Jensen-Smith et al., 2003; Rezania et al., 2008; Minoura, 2017), possibly because of control systems like tissue-specific promoters and/or transcription factors. The specificity of action of the subset of different tubulin isoforms present in any given cell is still elusive and even the occurrence of functional co-polymerization of the coexisting isoforms has not been confirmed *in vivo* beyond any doubt.

With the emergence of the first tubulinopathies at the beginning of this century (Keays et al., 2007; Jaglin et al., 2009) and with the progress made in the understanding of the natural history of these diseases, we are aware now that the majority of the clinical manifestations of tubulin mutations affect primarily the nervous system (Chakraborti et al., 2016). This is well in accordance with the prominent role played by tubulins in brain development through neuronal genesis and migration, cortical organization and also in axon guidance (Breuss et al., 2017). It is worth specifying that tubulinopathies are, to this date, the pathological manifestation of mutations in the genes of a just a subset of tubulin genes: TUBB2A, TUBB2B, TUBB3, TUBG1, TUBA8, TUBB, TUBA3E, and TUBB4A. However, this list is expected to grow as more genetic diagnoses are run and new mutations are discovered.

Of the tubulin mutations affecting the nervous system, most cause cerebral malformations and induce either developmental or degenerative changes in different cell types. TUBB4A represents an exception, being it directly associated to hypo- and demyelination (Gonçalves et al., 2018) and showing a range of clinical and radiological manifestations which depend on the position of the mutated residue and, possibly, on the cell type involved (Curiel et al., 2017). Hypomyelination with atrophy of the basal ganglia and cerebellum (H-ABC) is the condition caused by some TUBB4A mutations that has the most profound effect on the central nervous system (CNS) white matter, causing deficient myelin formation and even myelin degeneration during childhood (Curiel et al., 2017).

For TUBB4A, as well as for the majority of other tubulinopathy-causing isoforms, the two major unanswered questions are: (1) how a mutation in microtubules could affect the physiology of the cell expressing it? And more specifically, what is the molecular mechanism that connects an amino acidic change to the disfunction of glial cells of the CNS, where the phenotypical effects of TUBB4A mutations are most tangible? (2) what are the possible genetic or pharmacological strategies that might retard, limit or cancel the effects of tubulin mutations? Despite the knowledge of the primordial cause, i.e., the mutations, and the downstream macroscopic effects, little is known about the molecular pathology and how it could be reverted. The recent description of the disorders caused by tubulin mutations has not allowed for the accumulation of enough knowledge derived from both the functional tests performed with mutated tubulins in vitro and structural analysis of mutated tubulins in silico.

An animal model of tubulinopathy would provide an integral representation of the pathology, from the cellular to the organ system level, with the additional advantage of being able to follow the natural history of the disease in a timespan much shorter than a human life. The mechanisms underlying the disease could be observed, studied and manipulated in a living physiopathological system, not limiting the observation to either fixed tissues or simplified models as cell lines. The manipulation of the model is especially crucial for the testing of pharmacological drugs that can be employed to ameliorate the conditions of the patients. For studies on complex diseases and pharmacology, the laboratory rat is the prominent model (Twigger et al., 2008).

In this article, we provided information of the myelin-defective *taiep* rat as a model of tubulinopathy. The mutant spontaneously originated from the inbreeding process to obtain a high-yawning Sprague-Dawley rat (Holmgren et al., 1989). The animal model suffers from a leucodystrophy due to an initial hypomyelination followed by a progressive demyelination correlated to an accumulation of microtubules in the oligodendrocytes (Duncan et al., 1992; Couve et al., 1997; Lunn et al., 1997). Importantly, *taiep* rats have a long lifespan reaching 18 to 24 months of age (Cortés et al., 2005). The strength of the present study lies in the possibility of documenting by magnetic resonance imaging (MRI) the progression of the disease in a living animal with a longitudinal study. The imaging results are put in relation with the mutated tubulin phenotype symptoms, the same way and with similar results of what is done with human patients (Ji et al., 2018; Joyal et al., 2019). The resonance imaging analysis not only allowed us to visualize how the *taiep* MRI phenotype shares most of the patterns that characterize the human H-ABC radiological phenotype, but it will also represent an ideal tool for monitoring the evolution of the disease once pharmacological or genetic therapies are designed. The changes in the properties of the nervous tissue were highlighted also with optical microscopy, where, by means of bright field imaging of unstained tissue, we could show how the optical density of the white matter of cerebellum and spinal cord is reduced early in development and decreases dramatically due to the progressive deterioration of myelin and despite some persistence of neural fibers.

## Materials and Methods

### Longitudinal Magnetic Resonance Imaging: T2-Weighted Images

High-resolution images of the brain of male rats were acquired at 1, 2, and 8 months using a Helium-cooled 7.0 T scanner (BRUKER PHARMASCAN 70/16, Billerica, MA, United States) equipped with a gradient set with Gmax = 760 mT/m. For the MRI scan, six *taiep* and four Sprague Dawley wild type (WT) rats were analyzed at the three time points. The animals were anaesthetized with isoflurane (Sofloran, PiSA). A 5% concentration dose was used for induction and 1–2% for maintenance of an adequate level of anesthesia. The body temperature was kept constant throughout the experiment using a thermoregulated water circulation system. The oxygenation level of the rats was monitored by a pulse oximeter. T2-weigthed images (T2-WI) were acquired with contiguous 0.8 mm sections in the coronal plane using a Rapid Acquisition with Refocused Echoes (RARE) sequence with the following parameters: repetition time (TR) of 2673.5 ms; echo time (TE) of 33 ms, field of view (FOV) 20 mm × 18 mm, matrix size 200 × 180 corresponding to an in-plane resolution of 0.1 mm × 0.1 mm. T2-weigthed images of sagittal sections were acquired using a sequence with a TR of 15 ms, TE 3 ms, FOV 100 mm^2^, an in-plane resolution of 0.208 × 0.208 mm^2^. All procedures described have been done in compliance with the Laws and Codes approved in the Seventh title of the Regulations of the General Law of Health regarding Health Research of the Mexican Government (NOM-062-ZOO-1999), and in accordance with the recommendations of the National Institutes of Health Guide for the Care and Use of Experimental Animals and were approved by the institutional committee of bioethics in research of the University of Guanajuato.

### Tissue Preparation and Image Acquisition

*Taiep* and WT rats aged 1, 3, 7, and 10 months were anesthetized with a mixture of ketamine/xylazine (0.125 and 5 mg/kg, respectively) and then decapitated using a guillotine. Brain, cerebellum, and spinal cord were quickly removed and cut into blocks of approximately 0.5 cm and fixed in 4% formaldehyde (Sigma-Aldrich, F8775) in PBS for 60 min at room temperature. For brain sections, hemi-brains were transferred to 30% sucrose, at 4°C overnight and embedded in Tissue Freezing Medium (Leica, 14020108926) for cryoprotection. Serial sections (40 μm) were cut with a cryotome Leica CM 1850 mounted on Ultra-StickGold Seal glass slides (Becton Dickinson, Mexico) and stored at −80°C. One hundred twenty-five micrometer sections of fixed cerebellum and spinal cord were cut using a vibrating-blade microtome (Leica VT1200).

The samples for bright field microscopy were observed with either an LSM 710 Zeiss confocal microscope, using a 25× oil-immersion objective, or a Cytation 5 cell imaging multi-mode reader (Biotek, VT, United States), with a 4× objective. Pseudo-bright field images were generated with residual 405 nm laser light captured in transmission by an external non-descanned detector (NDD) of the Zeiss microscope.

For fluorescence microscopy, cervical spinal cord, brain and cerebellar slices were blocked and permeabilized using a PBS solution with 33% goat serum and 10% triton X-100. The samples were then incubated overnight with the Anti-Neurofilament 200 (Sigma-Aldrich N4142) primary antibody. The next day the slices were exposed to the Goat anti-Rabbit, Alexa Fluor 488 secondary antibody (Thermo Fisher A-11070), FluoroMyelin (Thermo Fisher F34652) and DAPI (Thermo Fisher 62247), and mounted in Fluorescence Mounting Medium (Dako, S302380). The slides were imaged with an LSM 710 Zeiss confocal microscope, using a 25× or a 63× oil-immersion objective. Myelin content was normalized to the highest value in each SD experiment. Differences between groups were tested with a Student *T*-test for the CC and with the Wilcoxon–Mann–Whitney test for the spinal cord. Significant levels are indicated as ^∗^*p* < 0.05.

FIJI^1^ was used to convert and reconstruct the images, while the GIMP software^2^ was used for image processing.

### DNA Extraction, Primers, PCR and Sequencing

Using the Quick-DNA Miniprep Plus kit (ZYMO RESEARCH, D4068), genomic DNA was extracted from the tail of one sacrificed *taiep*, WT and Wistar rat.

The coding sequence of the *Tubb4a* gene of *Rattus norvegicus* (NC_005108) is organized into four exons (Figure 5A). In our PCR strategy, we amplified exons 2 and 3 together in a single amplicon, due to their small sizes and the fact that they are separated by only 129 bp. On the other hand, due to exon 4 larger size, it was amplified using two separate pairs of primers. The two portions amplified were named exon 4A and exon 4B. Forward and reverse primers were designed in the intronic regions, approximately 20 bases away from the exons (see Figure 5A and Table 1). For each amplicon, PCR reaction tubes with a total volume of 20 μL were prepared, with primers added to a final concentration of 200 nM. Platinum PCR SuperMix (ThermoFisher, 12532016) was used for the amplification.

**TABLE 1:** Sequence of primers used to amplify the *Tubb4a* gene.

**Exon**	**Sequence**
1	5′CCGGTTGACACCCGTCCATC3′
	5′CCATTTCCGAGCCCCTCTGC3′
2-3	5′CAATAACAACAGAAAGGACAATC3′
	5′GCACTTATAAAACCAAACGTGTG3′
4A	5′CATACCTACAAATTCAACCCTGTC3′
	5′CTCGGTACTGCTGGCTGCCC3′
4B	5′CGTTCCCCCGTCTCCACTTC3′
	5′GATTTGCTGGTGTGCCCCGG3′

The sequencing of the PCR products was performed with the Sanger method, using the same primers previously used in the PCR reactions.

## Results

### MRI Features of the *Taiep* Rats

**FIGURE 1:**
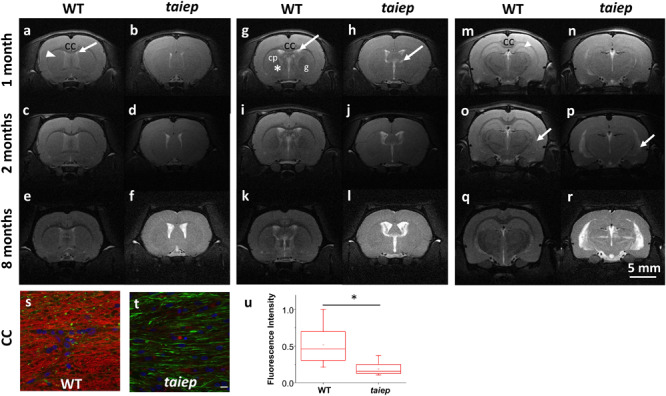
Representative MR T2-WI from one WT and one *taiep* rat brain. Coronal sections were taken at three different planes and time-points (1, 2, and 8 months). CC, striatum (arrow head), and lateral ventricles of WT and *taiep* are visible in the first plane **(a–f)**. In the second plane **(g–l)** is possible to discriminate the caudate-putamen (cp) from the globus pallidus (g) and the internal capsule (*) in control rats, while in *taiep* those structures are indistinguishable. In the third plane **(m–r)** the thalamus and the hippocampus are visible in the healthy rat (m), as well as a myelinated, hypointense hippocampal layer (arrow head). In the three planes the CC is thin or not visible, the ventricles of *taiep* rats are enlarged (arrows) and hyperintense signal is obtained from their surrounding areas. Six *taiep* and four WT rats were analyzed in parallel with equivalent results. **(s,t)** Confocal images of fluorescently labeled myelin (red), neurofilaments (green) and nuclei (blue) in the CC of 10 months WT **(s)** and *taiep* **(t)** rats. Scale 10 μm. **(u)** Normalized myelin fluorescence levels in the CC for 24 regions of interest drawn on eight images from eight rats (*p < 0.05, Student’s *t*-test).

Three coronal planes in the rostro-caudal axis were selected to optimally highlight the differences in recognizable anatomical structures between wild type and tubulin mutant rats using T2-WI (Figure 1 and Supplementary Table 1).

In the most rostral plane, the corpus callosum (CC) of *taiep* rats appears thinner than in WT rats at 1 month; thinning becomes more pronounced at 2 months, and at 8 months of age CC is no longer visible (see Figures 1b,d,f), while in WT rats it seems to thicken over time as expected (Figures 1a,c,e).

In the second plane (Figures 1g,i,k), WT rat brains exhibit distinctive white matter features at all ages. In this coronal section, the CC can be seen as a well-defined structure, whose low signal separates the neocortex (above) from the striatum (below). Caudate-putamen, globus pallidus (basal ganglia) and internal capsule (ic) are clearly defined in healthy rats at all evaluated ages (Figures 1g,i,k). When analyzing equivalent coronal sections of the *taiep* rats (Figures 1h,j,l), we found that the CC gets thinner, the ic is not visible and basal ganglia are poorly defined at any age.

The most caudal plane offers a comprehensive view of the CC, the hippocampus, the thalamus, the medial lemniscus (ML), the ic, and the ventricles of the WT rat (Figures 1m,o,q). Same as observed in more rostral planes, in the case of *taiep* rats the white matter is indistinguishable from the surrounding structures and, particularly in this plane, the CC is undetectable already since the first month of age (Figure 1n). While in the control animals the hippocampus is always clearly recognizable, its anatomical profile in the *taiep* rats is poorly identifiable (Figure 1p). In *taiep* rats the fimbria (fi) is not visible and, at 2 months, two out of six rats already presented hyperintensities where this structure should be located. There is also atrophy of the basal ganglia, with poor definition of the areas otherwise occupied by the striatum (Figures 1p,r) as well as hyperintensity of the ventricular system (arrows), which worsens with the age of the subjects. The signal from the ML of *taiep* rats is undetectable at 1 and 2 months but at 8 months the region of the ML gives hyperintense signal opposite to the low signal of ML in WT rats of any age.

**FIGURE 2:**
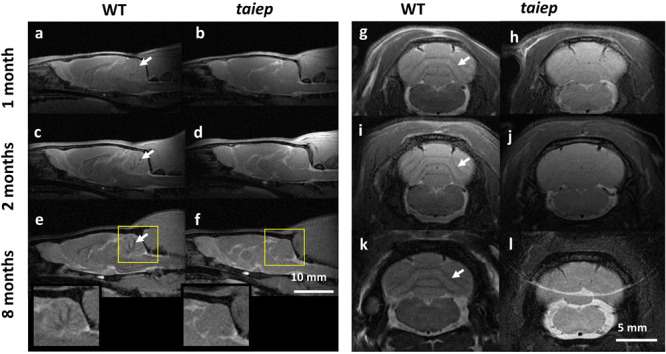
MR T_2_-WI of one *taiep* and one WT cerebellum. Sagittal **(a–f)** and a coronal **(g–l)** planes of cerebellum were imaged at 1, 2, and 8 months; **(e,f)** also include the zoom of the sagittal section at 8 months. The dark signal (arrows) in the coronal views of WT white matter pathways **(g,i,k)** is not visible in the corresponding images of the mutant **(h,j,l)**. Six *taiep* and four WT rats were analyzed in parallel with equivalent results.

T2-weighted images of cerebellum in *taiep* rats showed diffused atrophy (see Figure 2). Sagittal (Figures 2a–f) and coronal (Figures 2g–l) planes were included to help the visualization of the anomalous high signal of cerebellar white matter. In both planes the difference between healthy and mutant rats is evident even from the first month, suggesting that cerebellum is especially affected by demyelination.

The sagittal images include the whole brain where the CC and the ventricular system are clearly visible, corroborating from another projection plane the high signal from the CC and the enlargement of the ventricles (Figures 2a–f). At 2 months, an area of hyperintensity around the ventricles is also evident in two out of six mutant rats; at 4 months it can be detected in four rats and at 8 months all the animals presented this periventricular hyperintensity, whereas in WT rats this signal does not appear (Figure 1).

### Optical Microscopy Features of Cerebellum and Spinal Cord From the *Taiep* Rat

A consistent difference in optical density can be detected by eye when sectioning the cerebellum and spinal cord of *taiep* and WT rats. To convey this information and identify the source of the contrast difference at tissue level, bright field micrographs of entire unstained parasagittal sections were generated by stitching together partial images of the cerebellum taken with a low magnification objective.

**FIGURE 3:**
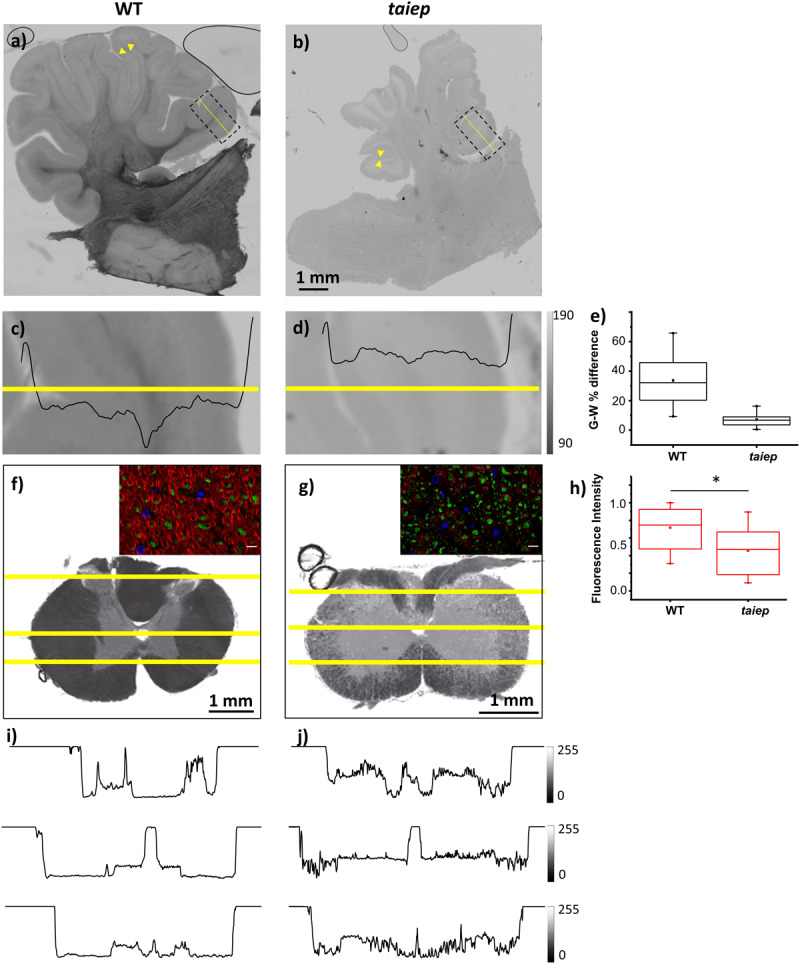
Optical contrast and fluorescence microscopy in CNS sections of 10 months WT and *taiep* rats. **(a,b)** Parasagittal section of cerebellum with a portion of brainstem; inbox: representative region of a folium. Arrowheads show the granule cell and molecular layers. **(c,d)** Image corresponding to the box drawn in **(a,b)**, with line graph of gray levels superimposed. Two WT and two *taiep* rats were analyzed. **(e)** Gray matter (G) – white matter (W) percent difference in gray values from the line-scans in panels **(c,d)** and Supplementary Table 2. **(f,g)** Transversal sections of cervical spinal cord. **(i,j)** Line graphs from the numbered lines drawn in panels **(f,g)**, showing gray level changes throughout the section. Three WT and three taiep rats were analyzed. Color insets in panels **(f,g)** correspond to the ventral funiculus labeled for myelin (red), neurofilaments (green) and nuclei (blue), scale 10 μm. **(h)** Normalized myelin fluorescence levels in the SC for 36 regions of interest drawn on 12 images from four rats (*p < 0.05, Wilcoxon–Mann–Whitney test).

The cerebella of the animals that underwent MRI analysis were sectioned for observation. In *taiep* rats the white matter regions of the sections appear more transparent to white light and do not generate optical contrast with the more peripheral layers of each folium, so the brightness of the entire section appears very homogeneous (Figures 3b,d), compared to similar regions of same age WT rats (Figures 3a,c); this depends on a change in composition and/or density of the tissue in those regions, very likely a lower amount of myelin. Conversely, the WT displays drastic changes in gray levels of white matter vs. gray matter (up to 65% difference in the folia, see Figure 3e and Supplementary Table 2.). Despite the change in brightness of the white matter portion in the images, the structure of the entire cerebellar cortex maintains its histological organization, e.g., the granule cell and molecular layers are clearly recognizable (Figures 3a,b, arrowheads).

Sections of mutant spinal cord observed with bright field microscopy at 10-months showed changes in the optical properties of the white matter which absorbed much less light compared to spinal cord sections from age-matched controls (Figures 3f,g). Moreover, the white matter optical density is homogeneous in WT rats while in *taiep* rats is non-homogeneous (Figures 3i,j).

Fluorescent microscopy of sections of the CC (Figures 1s,t) and of the SC (Figures 3f,g) confirms that the high intensity of the T2-weighted signal recorded by MRI in these regions, together with the reduction in optical density, can be related to a change, i.e., a reduction, in the myelination level, while neurofilaments persist in the same regions as confirmed by fluorescence microscopy (Figures 3f,g insets and 3h).

**FIGURE 4:**
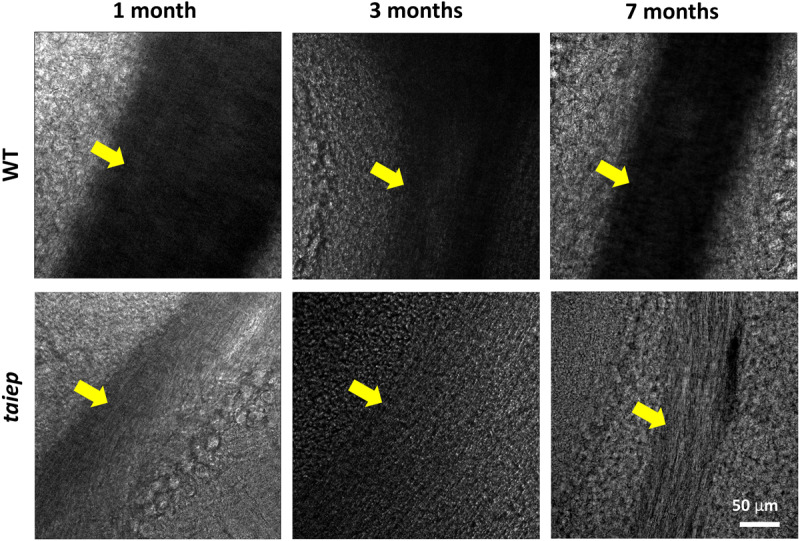
Pseudo-brightfield micrographs of cerebellum from three *taiep* and three WT rats. The parasagittal sections were imaged at different ages (1, 3, and 7 months). White matter generates optical contrast in the cerebellum of *taiep* at 1 month, although in a reduced manner, suggesting hypomyelination; at 7 months, *taiep* shows a remarkable reduction in optical density, not related to the white matter tract size and attributable to further loss of myelin. The yellow arrows indicate the region of the white matter.

To observe the phenomenon at a different scale, pseudo-bright field images of unstained cerebellar parasagittal slices from *taiep* and WT rats confirmed that in *taiep*, white matter is optically less dense, in this case to 405 nm light, compared to WT white matter, and this can be appreciated at all timepoints, suggesting that hypomyelination was present in the younger rats and demyelination took place afterward (Figure 4).

### Mutation in the *Tubb4a* Gene of the *Taiep* Rat and Implications on the Structure of the Microtubule

In the study by Li et al. (2003), the location of the gene responsible for the *taiep* phenotype was assigned to a subtelomeric region on chromosome 9. According to this genetic map, constructed thanks to the frequencies of recombination of several markers on chromosome 9, the possible mutation is in a region of 5.9 cM between the telomere and D9Rat88.

The conversion factors reported in Jensen-Seaman (2004) allowed us to estimate the base pair size of the region where the mutation associated with the *taiep* phenotype should be located. The physical size for chromosome 9 is 109.5 Mb, which corresponds to an approximate genetic distance of 78.3 cM for this rat chromosome. Based on this, it was possible to calculate the physical size of the 5.9 cM-region of interest. According to our approximation, the region measures 8250 kb and contains at least 130 genes. Some of them are associated with G proteins, others are related to ATP transmembrane transporters activity or have a Golgi-related activity. While reviewing each gene from this list, *Tubb4a* emerged as an interesting candidate: more than 30 mutations have been found in the *TUBB4A* gene and many of those cause abnormalities of the white matter. A group of mutations in this gene affects the oligodendrocytes causing hypomyelination; in addition, they are believed to alter the stability of the microtubules. Since these characteristics are similar to those that occur in *taiep*, the mutation causing the *taiep* phenotype was searched inside the *Tubb4a* gene.

The sequence analysis of the *taiep* genomic DNA was run in parallel with the analysis of wild-type Sprague Dawley DNA (WT) and also of Wistar DNA, to make sure all possible changes were going to be detected, including those which were characteristic of the WT background and/or the specific laboratory breed. The coding sequence of the WT and Wistar *Tubb4a* genes were identical and coincided with the sequence of Rattus norvegicus found in the NCBI database (NC_005108). Due to the design of the primers, we can also estimate that there is at least 6% identity of the intronic regions of the gene.

**FIGURE 5:**
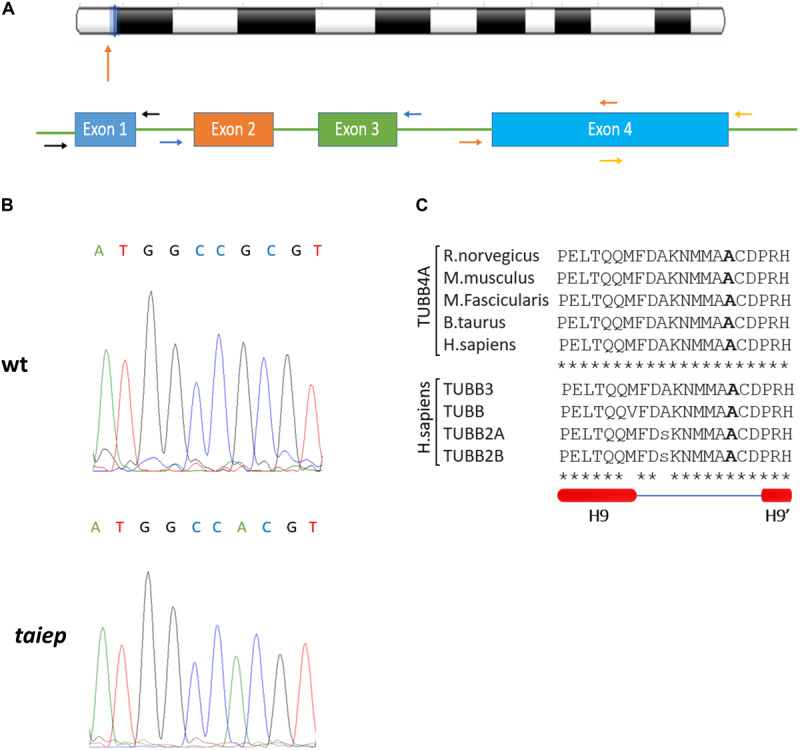
The *taiep* mutation. **(A)** Chromosome position and gene organization of the *Tubb4a* gene in *R. norvegicus*. **(B)** Electropherograms from WT and *taiep* rat. A G to A mutation was found in exon 4B of the *taiep* rat. **(C)** Above: portion of the amino acid sequence of the rat (NP_543158), mouse (NP_033477), macaque (NP_001271111) bovine (XP_024850289.1), and human (NP_006078.2) TUBB4A protein showing the mutated amino acid found in *taiep* (in bold). Centre: portion of the amino acid sequence of TUBB4A containing the *taiep* mutation aligned with other beta tubulins involved in tubulinopathies. Below: schematic representation of the secondary structure of the portion of tubulin sequence aligned above, according to Löwe et al. (2001). One *taiep*, one SD, and one Wistar rat were sequenced.

By comparing the sequences of *taiep* with WT and Wistar, we found a G to A point mutation in the exon 4B of the *taiep Tubb4a* gene (g.6337G>A, see Figure 5B). At the amino acid level, the *taiep* mutation changes an Ala by a Thr at position 302.

**FIGURE 6:**
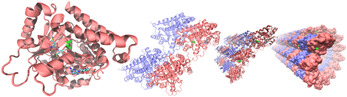
Structural context of Ala302 in the TUBB4A protein. From **(left)** to **(right)**: microtubules are built by the association tubulin alpha (blue) and beta isoforms (red). In the quaternary context, Ala302 (green) lies in a cleft along the microtubule separated more than a nanometer from any neighboring protein. In a single tubulin beta **(left panel)**, Ala302 is located in a loop region, solvent exposed, at the bottom of a shallow pocket. Methionines 299 and 300, the only two amino acids in the neighborhood reported as pathogenic, are shown colored by atom in glossy and pale colors, respectively. GTP within its binding site is also shown at the bottom of the right panel as a reference.

From the alignments of the amino acid sequences of macaque, cow, mouse, rat and human TUBB4A proteins emerges that the mutation found in *taiep* lies in a conserved interspecies site (Figure 5C). The same residue is also conserved in all beta tubulin isoforms (Tischfield and Engle, 2010), confirming the importance of the position for the proper functioning of the polymer. From the structural point of view, the possible effects of the Ala302Thr mutation are quite intriguing. Position 302 lies on the surface of TUBB4A on a non-structured region as shown in Figure 6. In stark contrast with other pathogenic mutations reported in the literature, Ala302 is located in the cleft within the microtubule, away from any protein-protein interface and is not in the close neighborhood of the catalytic site. Moreover, the relatively mild character of the mutation makes it difficult to foresee significant structural changes upon substitution of Ala302 with Thr.

## Discussion

The purpose of this work was to present the first animal model for H-ABC tubulinopathy. With what was known from the literature, i.e., a tubulin-related leukodystrophy was causing the *taiep* motor phenotype, we formulated the hypothesis that this Sprague Dawley mutant could display the MRI features that are indeed used for the clinical diagnosis of H-ABC. Therefore, by combining MRI, genetics and optical microscopy, we were able to prove that the specific MRI features of H-ABC were recognizable in a model where a point mutation in *Tubb4a* causes such a dramatic effect in the CNS that its change could be assessed with naked eye. Moreover, we were able to show with longitudinal MRI that the scarcity of myelin at birth is followed by progressive loss of white matter, making this murine model a faithful representation also of the progression of the myelin impairment. The structures affected by the tubulinopathy-dependent hypomyelination/demyelination coincide with those affected in the patients and also can be detected with the same approach that allows follow-up at different times, for example during pharmacological and/or immunological treatments. Hypomyelination with atrophy of the basal ganglia and cerebellum patients have developmental and degenerative problems caused by insufficient myelination and their diagnosis is not possible without resonance imaging; MRI from *taiep* rats show reduced myelinization already at one month of age and progressive further loss of the lipid insulator, causing atrophy of those regions where myelin is still present in control rats. Comparably, (Eguibar et al., 2012) showed a significant decrease in the lipid content in various structures of the CNS of *taiep* rats of 8 months of age with respect to Sprague-Dawley. The spatiotemporal pattern of myelination in rat, which begins around birth and proceeds in a caudal-to-rostral direction, reaches completion in the cerebellum at around 1 month (van Tilborg et al., 2018), although rates for oligodendrocytes turn-over are rather high for the whole life of control rats (Gibson et al., 2014).

The helium cooled high magnetic induction scanner (7 T) permitted us to obtain higher quality images than common clinical scanners which usually work at 3 T or less. The brain anatomy of *taiep* rats is deeply altered by the demyelination process. T2-weighted images from the mutant rat brain show high and homogeneous signal across white and gray matters. The corpus callosum and the corticofugal paths are composed of myelinated fibers which typically confer these organs a low signal for T2-WI in healthy individuals, but in *taiep* rat brains, the white matter tracts are almost indistinguishable from their surrounding structures. High intensity in the MRI signal is a sign of demyelination because as the myelin sheets degenerate, the space once occupied by myelin phospho- and glycolipids is now filled with cerebrospinal fluid and the signal generated from the atrophied fibers becomes more intense as the degeneration progresses.

The caudate-putamen and the globus pallidus of *taiep* rats are indistinguishable at 1, 2 and 8 months, while images of the control rats of the same age allow the differentiation of those areas. The corpus callosum, as well as the internal capsule are formed by highly myelinated fibers and therefore would be expected to generate dark areas in T2-WI, as is the case for control rats; in the tubulin mutants, they display high signal. Fluorescence staining of myelin in the CC confirms the reduction in the myelin content in the taiep rat, with persistence of some neural fibers. Moreover, as it is often the case with H-ABC patients, MRI hyperintensity is associated to enlarged ventriculi in *taiep* rats that could be a consequence of hydrocephalus ex vacuo. Hyperintensity is also detected around the ventricles in T2-WI (Figures 1p,r). This recalls periventricular leukomalacia (PVL), and even though it is not possible to identify cystic lesions at any age, there is indeed ventriculomegaly, loss of periventricular white matter and thinning of the CC, typical signs of PVL, which can also be found in some H-ABC patients (Hernandez et al., in preparation). Periventricular leukomalacia is usually related to prematurity, which is not the case in many of the H-ABC patients or in our tubulin mutant rat model, so we cannot conclude that these last radiographic features correspond to a PVL. Nevertheless, it is striking that PVL is associated with a failure of pre-OL differentiation and as a result hypomyelination (Volpe et al., 2011). These evidences point in the direction of a common phase for the two diseases during in utero development, in which the damage suffered by pre-oligodendrocytes is caused by hypoxia in PVL, and by tubulin malfunction in H-ABC and manifests itself as deficient myelination around the ventricles and further demyelination during the early life of the patients and also in maturing *taiep* rats, as demonstrated in this study.

Another white matter structure that shows demyelination in MRI of *taiep* rats is the medial lemniscus which displays high T2 signal during the first 2 months and is hyperintense at 8 months. The ML is an ascending white matter pathway formed by myelinated axons of second order neurons located in the nuclei gracilis and cuneatus, respectively, hence the low signal in WT rats. The loss of myelin in the corticospinal tract and fasciculus gracilis of *taiep* rats was previously described (Duncan et al., 1992). It was observed that in 2 months mutant rats the fasciculus gracilis and corticospinal tract are partially myelinated and by 12 months there is no myelin present in these areas. The MRI indicates that the demyelination observed in the spinal cord (Duncan et al., 1992; Couve et al., 1997; Lunn et al., 1997) is maintained along the corresponding pathways, such as the cortico-spinal tract.

In our MRIs, a distinct thin, dark line seen at all ages in the hippocampus of healthy rats corresponds to a myelinated layer, very likely in the dentate gyrus, however, in *taiep* rats it was recognizable only at one month (Figure 1m, arrowhead). This particular difference could indicate degradation of neurons near this area. Other studies confirm the presence of morphological (Silva-Gómez et al., 2018) and physiological (Fuenzalida et al., 2009) changes in the *taiep* hippocampus.

The cerebellum of *taiep* rats is atrophic losing all the characteristic patterns of white matter at all analyzed ages. The atrophy was also corroborated by T2-WI of sagittal sections (localizers), where the signal from cerebellar white matter is also homogeneous with respect to the surrounding cerebellar gray matter.

Having determined the feasibility of MRI to detect and follow in time the macrostructural changes associated to H-ABC opens the way to many more MRI applications, including some to deepen the anatomical knowledge of the affected CNS, like MRI diffusion tensor imaging (Concha et al., 2009) or techniques that could quantitatively estimate the change in myelination levels (Laule et al., 2004), and others that may help to understand the impact of the anatomical changes on brain physiology and even on social behavior (Serafini et al., 2016; Nat Neurosci Editorial, 2017).

Magnetic resonance imaging of rat CNS, at least performed with these parameters, does not allow us to determine if the size of cerebellum changes, but previous studies showed that it is one of the most affected areas (Song et al., 2001; Leon-Chavez et al., 2006). On the other hand, a slight reduction in size of *taiep* cerebellum could be qualitatively inferred by the optical microscopy images taken at low magnification from the animals that underwent MRI analysis. This smaller size, although visible in all cerebellar sections, is not as dramatic as the reduction in size present in H-ABC patients and could also derive from the moderate separation of the gyri, which in turn could be an effect of the myelin loss. While the architecture of the cerebellum did not display dramatic changes in terms of the constituent regions i.e., the distribution of the molecular, granule cells and Purkinje cells layers around the white matter, the optical density of the white tracts appears drastically reduced due to the reduced content in myelin. Higher magnification images taken from younger subjects confirm that the scarce contrast stems from both hypomyelination and demyelination. Light polarization effects in these images were minimized by placing the analyzed white matter tracts in the same orientation under the microscope. At least some of the neural fibers in the white matter persist in the demyelinating regions, indicating that the symptoms arise from the neuronal misfunction and damage caused by the loss (Curiel et al., 2017) of the insulating layer, but not directly from neural loss.

The optical density of the white matter in the spinal cord of *taiep* is also reduced and it is drastically less homogeneous when compared to images of the same regions of control rats. Micrographs from white matter tracts display small bright zones in between the otherwise dense, dark looking myelinated fiber tracts, pointing again toward a scattered loss of myelin.

The mutations found so far in the TUBB4A gene seem to have different effects on cell physiology. The mutations that cause dystonia type 4 (for example, p.Arg2Gly) alter neuronal morphology, but not that of oligodendrocytes, giving no detectable MRI phenotype (Curiel et al., 2017). The mutations that cause isolated hypomyelination and childhood encephalopathy damage the oligodendrocytes but do not affect neuronal morphology (Curiel et al., 2017). The mutation p.Asp249Asn, the most frequently occurring among the mutations that cause H-ABC, is the only one that could alter the morphologies of both oligodendrocytes and neurons, at least *in vitro*. All mutations in the TUBB4A gene alter the stability of microtubules or their interaction with motor or accessory proteins (Curiel et al., 2017). In this work we corroborate the genetic defect that may be the only responsible for the *taiep* phenotype (Duncan et al., 2017): the missense, point mutation in position 302 lies in a loop following H9. The mutated alanine residue is conserved among beta tubulins and also among many different organisms, like macaque, cow, mouse, rat, and human (Figure 5C). Only one spontaneous mutation is known to occur in this position in TUBB3 and it is associated with the ocular motility disorder CFEOM3 (Tischfield et al., 2010). The mutation, which is not among the most frequently occurring CFEOM3-causing mutations, stabilizes tubulin by reducing microtubule dynamics. The reason behind this stabilization has been related to the lateral interactions among protofilaments (Tischfield et al., 2010). However, due to the tridimensional arrangement of the protofilaments in a microtubule, residue 302 cannot be involved in molecular interaction with residues on the adjacent beta or alpha tubulin, due to the distance across the cleft imposed by the angular distribution of the protofilaments in a stable microtubule. Although the distances between adjacent copies of TUBB4A may be reduced in the intermediate states during microtubule assembly, when protofilaments lie in a sheet (Nogales and Wang, 2006), the possible structural implications are uncertain at this stage. Additionally, it may be worth mentioning that Withaferin A has been reported to bind to TUBB4A onto a binding pocket formed by Ala302, mediating growth arrest in human breast cancer cells (Antony et al., 2014). Given the peripheral location of this binding pocket, it is possible to speculate that Ala302 might take part in a protein-protein interface with a still undetermined partner, yet this type of interactions could be investigated in further studies.

Finally, the analysis of the residues that lie in close proximity to amino acid 302 in the 3D structure of TUBB4A (299–305, 373–380, 201–208) shows that only two positions have been already associated to human mutations, namely Met299 and Met300. In contrast to Ala302, both Methionine residues are buried in the protein, forming part of its hydrophobic core (see Figure 6). The M299L variant has been reported, although with no additional information about its likely damaging effects on the protein structure/function [National Center for Biotechnology Information. ClinVar; (VCV000429972.2)]^3^. For position 300, on the other hand, a quite clear evidence of the pathogenic effects of the M300I mutation is present in the literature for four patients so far. Their clinical symptoms include delayed motor and speech development, with progressive deterioration of the motor function, including ataxia, dysarthria or anarthria (Erro et al., 2015; Pyle et al., 2015). MRIs of these patients reveal the most common feature of H-ABC: hypomyelination/demyelinization with atrophy of the cerebellum. With respect to the atrophy of the neostriatum, the reduction in size of the putamen and the caudate nucleus is common to all but one of the patients analyzed, confirming that MRI features associated to this mutation stand at the most severe end of the spectrum. This is an additional indication that changes in amino acids in close proximity to the *taiep* mutation cause the classic clinical and radiological H-ABC phenotypes in human patients and therefore it confirms that the *taiep* rat is indeed a valid model for the study of this tubulinopathy.

## Data Availability Statement

The raw data supporting the conclusions of this article will be made available by the authors, without undue reservation, to any qualified researcher.

## Ethics Statement

The animal study was reviewed and approved by Institutional committee of bioethics in research of the University of Guanajuato.

## Author Contributions

VP and VH conceived the work. AG-R performed and analyzed the molecular biology and optical microscopy experiments. MA performed and analyzed the MRI experiments. SP contributed structural analysis. VP, VH, MA, AG-R, and SP wrote the manuscript. VP, VH, MA, AG-R, SP, JE, and CC revised the manuscript.

## Conflict of Interest

VP and VH are members of the same family.

The remaining authors declare that the research was conducted in the absence of any commercial or financial relationships that could be construed as a potential conflict of interest.

## Footnotes

1 https://fiji.sc

2 https://www.gimp.org

3 https://www.ncbi.nlm.nih.gov/clinvar/variation/VCV000429972.2, accessed October 15, 2019.
