# Supplementary material for: MRI Features in a Rat Model of H-ABC Tubulinopathy
Source: Front Neurosci. 2020 Jun 3;14:555. doi: 10.3389/fnins.2020.00555 (PMC7284052; doi:10.3389/fnins.2020.00555)
Supplement: Supplementary file 1 [file Table_1.docx]

| Rat/feature | taiep 1 | | taiep 2 | | taiep 3 | | taiep 4 | | taiep 5 | | taiep 6 | | SD 1 | | SD2 | | SD 3 | | SD 4 | |
| --- | --- | --- | --- | --- | --- | --- | --- | --- | --- | --- | --- | --- | --- | --- | --- | --- | --- | --- | --- | --- |
| Thinner corpus callosum | 1 mo | Very thin | 1 mo | Not visible | 1 mo | Very thin | 1 mo | Very thin | 1 mo | Not visible | 1 mo | Very thin | 1 mo | thick | 1 mo | thick | 1 mo | thick | 1 mo | thick |
|  | 2 mo | Thin | 2 mo | Very thin | 2 mo | Not visible | 2 mo | Very thin | 2 mo | Very thin | 2 mo | Very thin | 2 mo | thicker | 2 mo | thicker | 2 mo | thicker | 2 mo | thicker |
|  | 8 mo | Very thin | 8 mo | Not visible | 8 mo | Not visible | 8 mo | Not visible | 8 mo | Not visible | 8 mo | Very thin | 8 mo | NA | 8 mo | NA | 8 mo | NA | 8 mo | thicker |
| Well delimited basal ganglia | 1 mo | no | 1 mo | no | 1 mo | no | 1 mo | no | 1 mo | no | 1 mo | no | 1 mo | yes | 1 mo | yes | 1 mo | yes | 1 mo | yes |
|  | 2 mo | no | 2 mo | no | 2 mo | no | 2 mo | no | 2 mo | no | 2 mo | no | 2 mo | yes | 2 mo | yes | 2 mo | yes | 2 mo | yes |
|  | 8 mo | no | 8 mo | no | 8 mo | no | 8 mo | no | 8 mo | no | 8 mo | no | 8 mo | NA | 8 mo | NA | 8 mo | yes | 8 mo | yes |
| Internal capsule is visible | 1 mo | no | 1 mo | no | 1 mo | no | 1 mo | no | 1 mo | no | 1 mo | no | 1 mo | yes | 1 mo | yes | 1 mo | yes | 1 mo | yes |
|  | 2 mo | no | 2 mo | no | 2 mo | no | 2 mo | no | 2 mo | no | 2 mo | no | 2 mo | yes | 2 mo | yes | 2 mo | yes | 2 mo | yes |
|  | 8 mo | no | 8 mo | no | 8 mo | no | 8 mo | no | 8 mo | no | 8 mo | no | 8 mo | NA | 8 mo | NA | 8 mo | yes | 8 mo | yes |
| Medial lemniiscus typical low signal | 1 mo | Very diffuse | 1 mo | diffuse | 1 mo | diffuse | 1 mo | diffuse | 1 mo | Very diffuse | 1 mo | diffuse | 1 mo | yes | 1 mo | yes | 1 mo | yes | 1 mo | yes |
|  | 2 mo | Very diffuse | 2 mo | diffuse | 2 mo | diffuse | 2 mo | diffuse | 2 mo | diffuse | 2 mo | Very diffuse | 2 mo | yes | 2 mo | yes | 2 mo | yes | 2 mo | yes |
|  | 8 mo | artifact | 8 mo | artifact | 8 mo | high signal | 8 mo | artifact | 8 mo | high signal | 8 mo | high signal | 8 mo | NA | 8 mo | NA | 8 mo | yes | 8 mo | yes |
| Hippocampus anatomical profile | 1 mo | low | 1 mo | low | 1 mo | Very low | 1 mo | Very low | 1 mo | low | 1 mo | low | 1 mo | yes | 1 mo | yes | 1 mo | yes | 1 mo | yes |
|  | 2 mo | low | 2 mo | low | 2 mo | Very low | 2 mo | low | 2 mo | low | 2 mo | low | 2 mo | yes | 2 mo | yes | 2 mo | yes | 2 mo | yes |
|  | 8 mo | none | 8 mo | none | 8 mo | none | 8 mo | none | 8 mo | none | 8 mo | none | 8 mo | NA | 8 mo | NA | 8 mo | poco | 8 mo | yes |
| Hyperintensity around hippocampus | 1 mo | low | 1 mo | no | 1 mo | no | 1 mo | no | 1 mo | no | 1 mo | no | 1 mo | no | 1 mo | no | 1 mo | no | 1 mo | no |
|  | 2 mo | mild | 2 mo | mild | 2 mo | low | 2 mo | low | 2 mo | no | 2 mo | no | 2 mo | no | 2 mo | yes | 2 mo | no | 2 mo | no |
|  | 8 mo | pronounced | 8 mo | pronounced | 8 mo | pronounced | 8 mo | pronounced | 8 mo | pronounced | 8 mo | pronounced | 8 mo | NA | 8 mo | NA | 8 mo | no | 8 mo | no |
| Hyperintensity of the ventricular system | 1 mo | low | 1 mo | no | 1 mo | no | 1 mo | no | 1 mo | no | 1 mo | no | 1 mo | no | 1 mo | no | 1 mo | no | 1 mo | no |
|  | 2 mo | low | 2 mo | low | 2 mo | low | 2 mo | low | 2 mo | no | 2 mo | no | 2 mo | no | 2 mo | yes | 2 mo | no | 2 mo | no |
|  | 8 mo | pronounced | 8 mo | pronounced | 8 mo | pronounced | 8 mo | pronounced | 8 mo | pronounced | 8 mo | pronounced | 8 mo | NA | 8 mo | NA | 8 mo | no | 8 mo | no |
| Atrophy of cerebellum | 1 mo | mild | 1 mo | mild | 1 mo | mild | 1 mo | mild | 1 mo | pronounced | 1 mo | pronounced | 1 mo | no | 1 mo | no | 1 mo | no | 1 mo | no |
|  | 2 mo | pronounced | 2 mo | pronounced | 2 mo | pronounced | 2 mo | pronounced | 2 mo | pronounced | 2 mo | pronounced | 2 mo | no | 2 mo | no | 2 mo | no | 2 mo | no |
|  | 8 mo | pronounced | 8 mo | pronounced | 8 mo | pronounced | 8 mo | pronounced | 8 mo | pronounced | 8 mo | pronounced | 8 mo | NA | 8 mo | NA | 8 mo | no | 8 mo | no |
| hyperintensity around the ventricles | 1 mo | low | 1 mo | low | 1 mo | no | 1 mo | no | 1 mo | no | 1 mo | no | 1 mo | no | 1 mo | no | 1 mo | no | 1 mo | no |
|  | 2 mo | low | 2 mo | low | 2 mo | no | 2 mo | no | 2 mo | no | 2 mo | no | 2 mo | no | 2 mo | yes | 2 mo | no | 2 mo | no |
|  | 8 mo | yes | 8 mo | yes | 8 mo | yes | 8 mo | yes | 8 mo | yes | 8 mo | yes | 8 mo | NA | 8 mo | NA | 8 mo | no | 8 mo | no |

NA: not available

**Table 1**. MRI features per subject.
