# Supplementary material for: MRI Features in a Rat Model of H-ABC Tubulinopathy
Source: Front Neurosci. 2020 Jun 3;14:555. doi: 10.3389/fnins.2020.00555 (PMC7284052; doi:10.3389/fnins.2020.00555)
Supplement: Supplementary file 2 [file Table_2.docx]

| **Cerebellum section** | **White matter (avg. intensity values)** | **Grey matter (avg. intensity values)** | **Percent difference** |
| --- | --- | --- | --- |
| taiep 1 |  |  |  |
| Region 1 | 171.2141666 | 183.635612 | 6.76 |
| Region 2 | 165.6708207 | 180.66535 | 8.29 |
| Region 3 | 160.159139 | 167.3020726 | 4.27 |
| Region 4 | 162.380131 | 167.5851286 | 3.1 |
| Region 5 | 168.1658033 | 168.91403 | 0.45 |
| taiep 2 |  |  |  |
| Region 1 | 45527.34668 | 47237.25914 | 3.62 |
| Region 2 | 44132.80567 | 47759.70968 | 7.6 |
| Region 3 | 44840.35494 | 47504 | 5.6 |
| Region 4 | 43278.5327 | 44888.9821 | 3.59 |
| Region 5 | 43930.6519 | 47634.4011 | 7.78 |
| taiep 3 |  |  |  |
| Region 1 | 26464.3166 | 31614.0712 | 16.29 |
| Region 2 | 22018.4886 | 25143.3769 | 12.49 |
| Region 3 | 23540.3575 | 24979.756 | 5.77 |
| Region 4 | 20993.4082 | 24154.5621 | 13.09 |
| Region 5 | 24546.2432 | 26958.0125 | 8.95 |
| SD 1 |  |  |  |
| Region 1 | 74.0507621 | 126.096 | 41.28 |
| Region 2 | 79.62401 | 117.35155 | 32.15 |
| Region 3 | 96.0508633 | 122.390857 | 21.52 |
| Region 4 | 91.57684 | 126.130571 | 27.4 |
| Region 5 | 94.79863 | 117.128379 | 19.07 |
| SD 2 |  |  |  |
| Region 1 | 25232.2052 | 31435.7329 | 19.74 |
| Region 2 | 18836.4473 | 30817.085 | 38.88 |
| Region 3 | 24348.9861 | 31393.5295 | 22.44 |
| Region 4 | 26402.0908 | 29115.4313 | 9.32 |
| Region 5 | 25486.1348 | 31969.3082 | 20.28 |
| SD 3 |  |  |  |
| Region 1 | 14816.306 | 26956.5089 | 45.04 |
| Region 2 | 9107.428 | 26607.7894 | 65.78 |
| Region 3 | 14327.9105 | 28570.2515 | 49.85 |
| Region 4 | 15920.4551 | 29873.7262 | 46.71 |
| Region 5 | 11962.8302 | 22077.2914 | 45.81 |

**Table 2.** Percent difference in gray values from cerebellar images
